# Solitary Secondary Malignant Melanoma of Clavicle Two Years after Enuclation for Ocular Melanoma

**DOI:** 10.1155/2013/591679

**Published:** 2013-01-28

**Authors:** Halil Tozum, Korhan Ozkan, Krishna Reddy, Ismail Turkmen, Ufuk Ciloglu, Serkan Senol, Calogero Graci

**Affiliations:** ^1^Department of Thoracic Surgery, ISMU Göztepe Education and Research Hospital, Istanbul Medeniyet University, 34732 Istanbul, Turkey; ^2^Department of Orthopaedics and Traumatology, ISMU Göztepe Education and Research Hospital, Faculty of Medicine, Istanbul Medeniyet University, 34732 Istanbul, Turkey; ^3^Department of Musculoskeletal Oncology, The Royal Orthopaedic Hospital, Birmigham B31 2AP, UK; ^4^Department of Cardiovascular Surgery, Siyami Ersek Thoracic and Cardiovascular Surgery Center, Istanbul, Turkey; ^5^Department of Pathology, ISMU Göztepe Education and Research Hospital, Faculty of Medicine, Istanbul Medeniyet, 34732 Istanbul, Turkey; ^6^Department of Orthopaedics and Traumatology, Agostino Gemelli Hospital, Catholic University of the Sacred Heart, 00168 Rome, Italy

## Abstract

Solitary metastasis of uveal melanoma to bone is extremely rare and usually associated with other organ involvement. We present a rare case of an ocular melanoma patient presenting with solitary metastasis to the clavicle two years after enucleation, without any other organ involvement. In this report, we tried to present our treatment strategy for the solitary metastasis of bone.

## 1. Introduction

Malignant melanoma accounts for 1–3% of all malignancies with an increasing incidence being seen worldwide [1, 2]. Uveal melanoma (UM) arises from melanocytes located in the choroid layer between the sclera and the retina. Although it is the most common primary malignant primary tumor of the eye, there are only about 1,500 diagnoses per year in the United States [3, 4]. Despite optimal treatment (surgery or radiation), metastases often develop with a mean period of 24–48 months and usually are associated with poor survival [5, 6]. 

Metastasis from malignant melanoma is known to spread by local extension, by the lymphatics or by the bloodstream. Blood-borne distant metastases of melanoma are seen in the lungs, gastrointestinal tract, brain, parotid, heart, and skin [7–13]. Solitary metastasis of uveal melanoma to bone is extremely rare and usually associated with another organ involvement predominantly liver or lung [14, 15]. 

We present a rare case of an ocular melanoma patient presenting with solitary metastasis to the clavicle two years after enucleation, without any other organ involvement. 

## 2. Case Report

**Figure 1:**
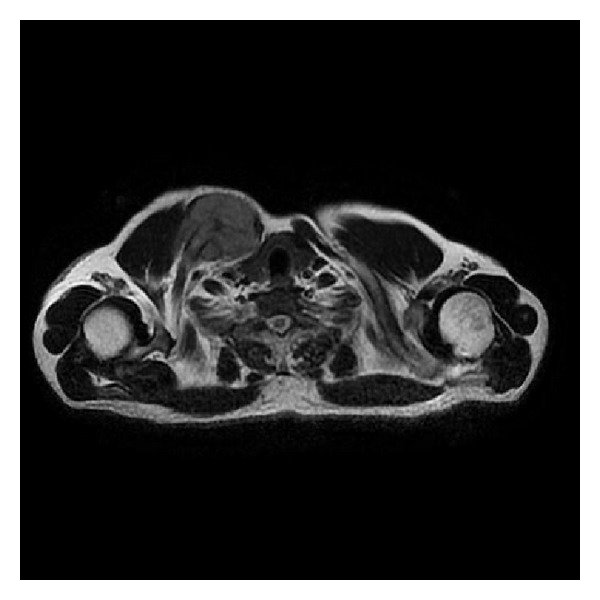
Axial MRI section with mass on the medial side of the clavicle.

**Figure 2:**
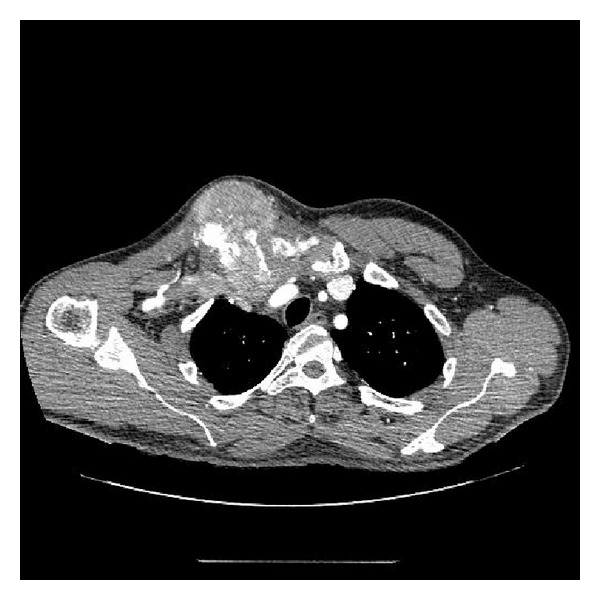
Axial CT section with mass on the medial side of the clavicle.

**Figure 3:**
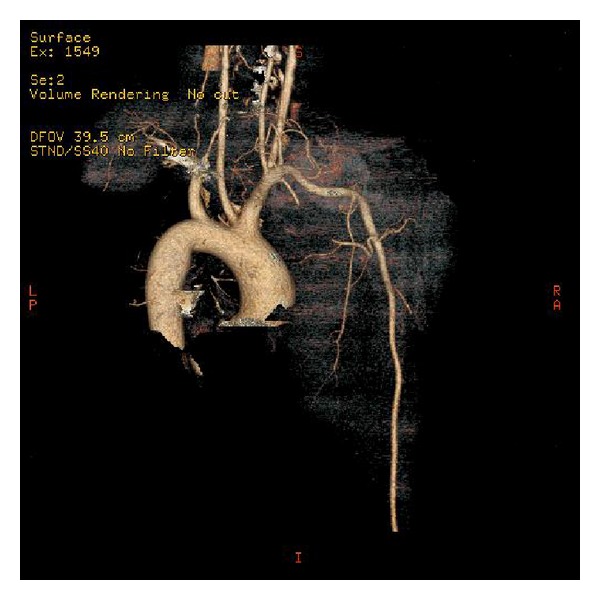
Preoperative CT-guided angiography displaying no vascular involvement but close proximity.

**Figure 4:**
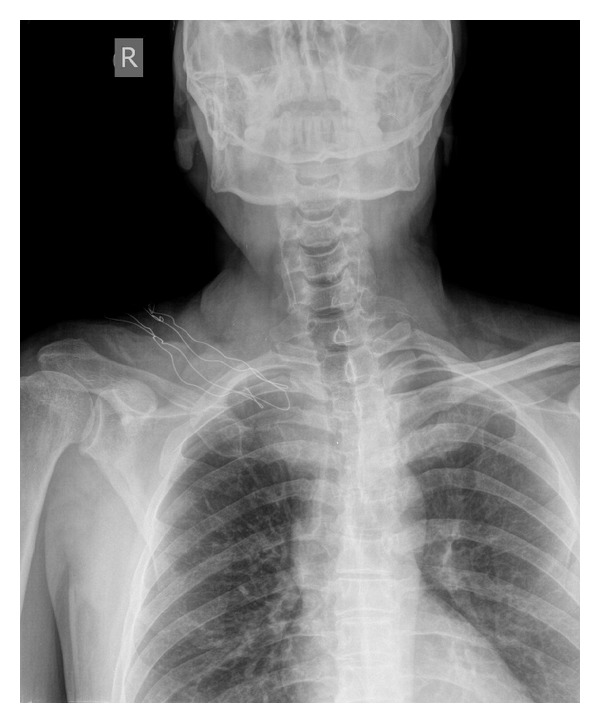
Anteroposterior roentgenography after removal of right clavicle.

**Figure 5:**
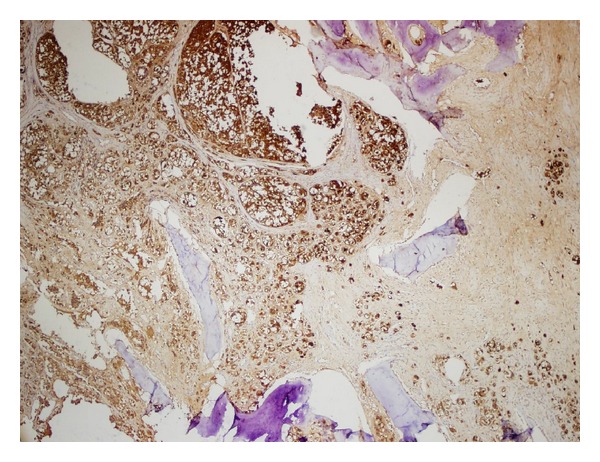
Strong Melan A immunoreactivity of metastatic tumor cells among the purple-colored bone cortex 1 × 100 magnification.

A thirty-four-year-old male patient presented to us with a two-month history of lump around his sternoclavicular joint. This was growing rapidly. Physical examination revealed a solid, nonmobile lesion on the medial end of the clavicle of approximately seven centimetre diameter. He was treated for uveal malignant melanoma two years ago (enucleation of left eye with cyberknife) at another centre. All haematological parameters and imaging were obtained which included magnetic resonance imaging (MR), computed tomography (CT), Nuclear bone scan, positron emission tomography (PET), and angiography (CT and Invasive). The tumour mass kept growing rapidly during this interval (two weeks), and a huge mass with a diameter of 13 cm and close proximity to innominate vessel on the medial side of clavicle was detected. Axial MRI section with mass on the medial side of the clavicle and Axial CT section with mass on the medial side of the clavicle (Figures 1 and 2). There was an increased uptake on bone scan at this site. PET scan showed this to be a solitary lesion. A CT-guided biopsy was performed. Biopsy was consistent malignant melanoma. An en bloc resection of the tumour along with the clavicle was planned. Preoperative embolization was carried out in view of the large feeder vessels identified on angiography. A transverse incision was made along the length of the clavicle, and care was taken to avoid any injury to the vessels and vagus nerve. The tumour was found to have an intrathoracic extension compressing on the innominate artery. Preoperative CT-guided angiography displaying no vascular involvement but close proximity (Figure 3). Postoperative recovery was uneventful. Anteroposterior roentgenography after removal of right clavicle (Figure 4). Strong Melan A immunoreactivity of metastatic tumor cells among the purple-colored bone cortex 1 × 100 magnification (Figure 5). He wore a sling for two weeks after which physical therapy was commenced. He was administered chemotherapy as per the oncologists advice, although its role in this situation is contentious. The patient also received radiotherapy after the healing of the surgical wounds. He regained full range of motion and function with one month after surgery. At his last followup (four months after surgery), he has been free of disease with no signs of local recurrence or disseminated disease with full function. 

## 3. Discussion

Over a 25-year period from 1973 to 1997, incidence of UM in the United State has been determined to be 4.3 cases per million people per year, which is similar to the report from European countries [16, 17]. Though UM is relatively rare compared with other malignant tumors, it contributes to a large proportion of deaths and leads to distant metastases, even after successful treatment of the local tumor [18, 19]. Soft tissue/lymph nodes, liver, lung, and brain are the commonest site of metastases. Skeletal metastasis is usually associated with end-stage disease along with disseminated disease in other organs concomitantly [14, 15, 20]. The treatment for metastatic bone lesion is to alleviate pain, prevent or treat pathological fractures, and decompress the pathological mass if causing spinal cord compression [21]. Wedin et al. stated that solitary skeletal metastasis in a melanoma patient without other manifestation of the disease in other organs might be an indication for radical resection to achieve a long-term survival [22]. 

Patients with claviculectomy function well without any symptoms [23]. A decision to remove the mass en bloc with all the clavicle instead of leaving a lateral stump was made to avoid possible pain at the free end of bone and skin problems due to compression by the stump. Patients with history of previous malignant melanoma should be alert and seek early consultation should they experience any pain and swelling that does not subside. A high index of suspicion should be maintained regarding the possibility of metastatic disease in these situations. The role of a multidisciplinary approach cannot be over emphasized for oncological surgery especially when performed in unusual sites or situations. 

## 4. Conclusion

 Solitary skeletal metastasis other than vertebrae in malignant melanoma is rare and has favourable prognosis comparing to other organs involvement. Radical resection of bone with radiotherapy with or without chemotherapy in selected patients seems to be the best option for survival.
